# Inhibition of sperm motility in male macaques with EP055, a potential non-hormonal male contraceptive

**DOI:** 10.1371/journal.pone.0195953

**Published:** 2018-04-19

**Authors:** Michael G. O’Rand, Katherine G. Hamil, Tiffany Adevai, Mary Zelinski

**Affiliations:** 1 Department of Cell Biology & Physiology, University of North Carolina at Chapel Hill, Chapel Hill, North Carolina, United States of America; 2 Research and Development, Eppin Pharma Inc., Chapel Hill, North Carolina, United States of America; 3 Division of Reproductive & Developmental Sciences, Oregon National Primate Research Center, Beaverton, Oregon, United States of America; 4 Department of Obstetrics & Gynecology, Oregon Health & Science University, Portland, Oregon, United States of America; University Hospital of Münster, GERMANY

## Abstract

Men have two practical choices for contraception; the condom which has a high typical use failure rate or vasectomy. New male hormonal and non-hormonal contraceptives are under development that target either the production of sperm (spermatogenesis) or the delivery of sperm. One particular target is the sperm protein EPPIN, which is present on the surface of human spermatozoa. EP055 is a small organic compound that targets EPPIN on the surface of sperm and inhibits motility. EP055 was tested in cynomolgus (*Macaca fascicularis)* males to determine its plasma half-life after intravenous (i.v.) infusion of a single dose and for binding to its target tissues. Our initial study demonstrated a plasma half-life for EP055 of 10.6 minutes. In a second study examination of macaque testis, epididymis, and plasma after i.v. infusion of a single dose of compound EP055 (63.25 mg/kg) demonstrated that EP055 was detected in testis and epididymis two hours and six hours post-infusion. We initiated a trial in rhesus (*Macaca mulatta*) males to assess the availability of EP055 in semen and its effect on sperm motility as a measure of the drug's efficacy. Four macaques were infused with a low dose (75–80 mg/kg) followed by a recovery period and a subsequent high dose (125–130 mg/kg) of EP055. After high dose administration, sperm motility fell to approximately 20% of pretreatment levels within 6 hours post-infusion; no normal motility was observed at 30 hours post-infusion. Recovery of sperm motility was obvious by 78 hours post-infusion; with full recovery in all animals by 18 days post-infusion. EP055 has the potential to be a male contraceptive that would provide a reversible, short-lived pharmacological alternative.

## Introduction

Men have two practical choices for contraception; the condom which has a high typical use failure rate (18%) or vasectomy which is expensive and not readily reversible [1]. New male hormonal [2] and non-hormonal [3,4] contraceptives are under development, which may target either the production of sperm (spermatogenesis) or the delivery of sperm, through physical blockage [5,6] or loss of function [3].

One particular target of interest is the sperm surface protein EPPIN, whose function is three-fold; namely as a bactericide [7], an inhibitor of PSA enzyme activity [prostate specific antigen, a serine protease) [8] and a protein that binds the seminal plasma protein semenogelin (SEMG1)[9]. The binding of EPPIN by anti-EPPIN antibodies (a-EAb) or SEMG1 causes a loss of sperm function manifested as a loss of forward or progressive motility [10–12]. In a normal fertilization scenario, ejaculated sperm coated with SEMG1 undergo a temporary loss of forward motility that is regained after removal of SEMG1 by PSA. Failure to remove SEMG1 due to a lack of PSA activity results in seminal hyper-viscosity and infertility [13–16]. Anti-EPPIN antibodies bound to sperm substitute for SEMG1 and result in reversible infertility [12].

Accordingly, sperm surface EPPIN is a reasonable non-hormonal contraceptive target that warrants further study. Because an anti-EPPIN immuno-contraceptive is not considered a viable commercial option [3], other approaches have been explored, including the search for small organic compounds to substitute for SEMG1 or a-EAb, both of which have overlapping binding sites on the C-terminal domain of EPPIN. In order to optimize our lead compounds for binding to the C-terminal domain of EPPIN, we first characterized SEMG1’s binding to EPPIN and defined the minimal sequence necessary to inhibit human sperm motility [3,17]. We expect the EPPIN amino acid residues that interact with SEMG1 to be the critical ones to which our lead compound will bind in order to inhibit sperm motility. The functional consequence of either SEMG1 or a-EAb binding to sperm is a decrease in sperm internal pH and a decrease in internal Ca^2+^ levels [18]. Therefore, we have searched for small organic compounds that would substitute for SEMG1 or a-EAb and cause a loss of sperm function. Here we report the effects of one of our lead compounds *in vivo*, examining its presence in semen of male macaques and its effects on sperm motility.

## Results

**Fig 1:**
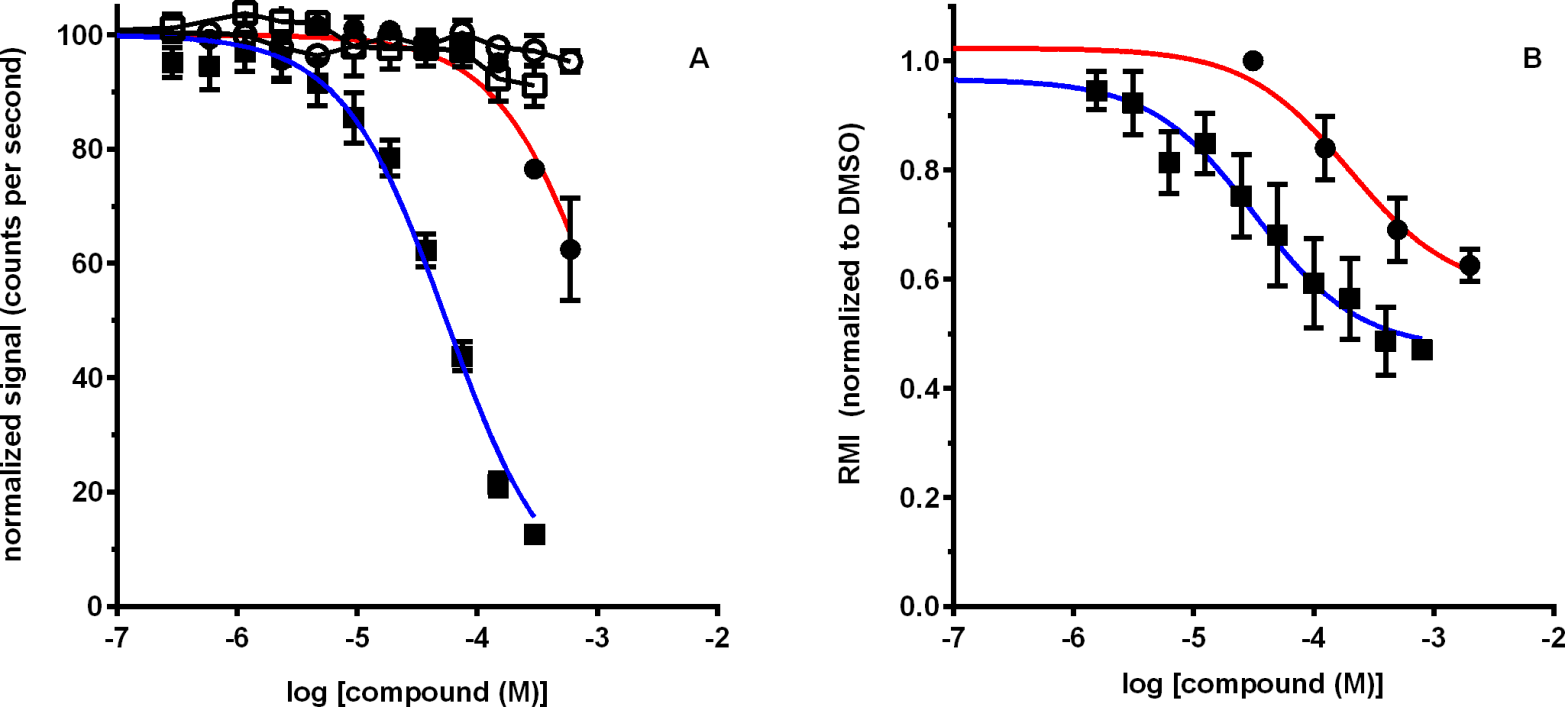
EP012 and EP055 interaction with Eppin. (A) AlphaScreen assay of a-EAb binding to EPPIN, closed symbols are AlphaScreen assay, open symbols are for non-specific interactions of compound with assay components. EP012 = box/blue line, EP055 = circle/red line. (B) CASA Assay showing the relative motility index (RMI = straight-line velocity (VSL) x % normal motility normalized to DMSO vehicle control). EP012 = box/blue line, EP055 = circle/red line. Curves represent the mean ± SEM of 4 human sperm donors.

During the course of developing new lead compounds that bind the protein EPPIN on the surface of human spermatozoa and inhibit motility, we assayed numerous small organic compounds that had multiple substitutions on a 1, 3, 5 triazine ring structure using AlphaScreen assays [3,17]. EP012, derived from the parent compound of this triazine ring group (S1 Fig), binds to the C-terminal domain of EPPIN as previously described for the SEMG1 peptide E2Q [17]. As shown in an AlphaScreen assay of a-EAb binding to EPPIN (Fig 1A), EP012 inhibits a-EAb from binding EPPIN with an IC_50_ of 55.4 μM +/-1.1 (n = 4). In a CASA (Computer Assisted Sperm Analysis) assay of human sperm (Fig 1B), EP012 inhibited sperm motility with an average relative motility index (RMI) IC_50_ of 33.4 μM +/-1.6 (n = 4 donors). Our preliminary testing in rats indicated a plasma half-life of less than 4 minutes.

Therefore, in an effort to improve the plasma half-life and solubility of EP012, derivatives of EP012, EP054 and EP055, were designed (S1 Fig). EP054 was completely hydrolyzed in plasma to EP055. Consequently, EP055 was used in further testing. As shown in an AlphaScreen assay of a-EAb binding to EPPIN (Fig 1A), EP055 inhibits a-EAb from binding EPPIN with an IC_50_ of 1121 μM +/-1.1 (n = 4). In a CASA assay using human sperm (Fig 1B), EP055 inhibited sperm motility with an average RMI IC_50_ of 199.5 μM+/-1.3 (n = 4 donors). The IC_50_ values for EP055 were not as low as EP012 values. However, the increased solubility and plasma half-life allowed *in vivo* testing of the compound.

**Fig 2:**
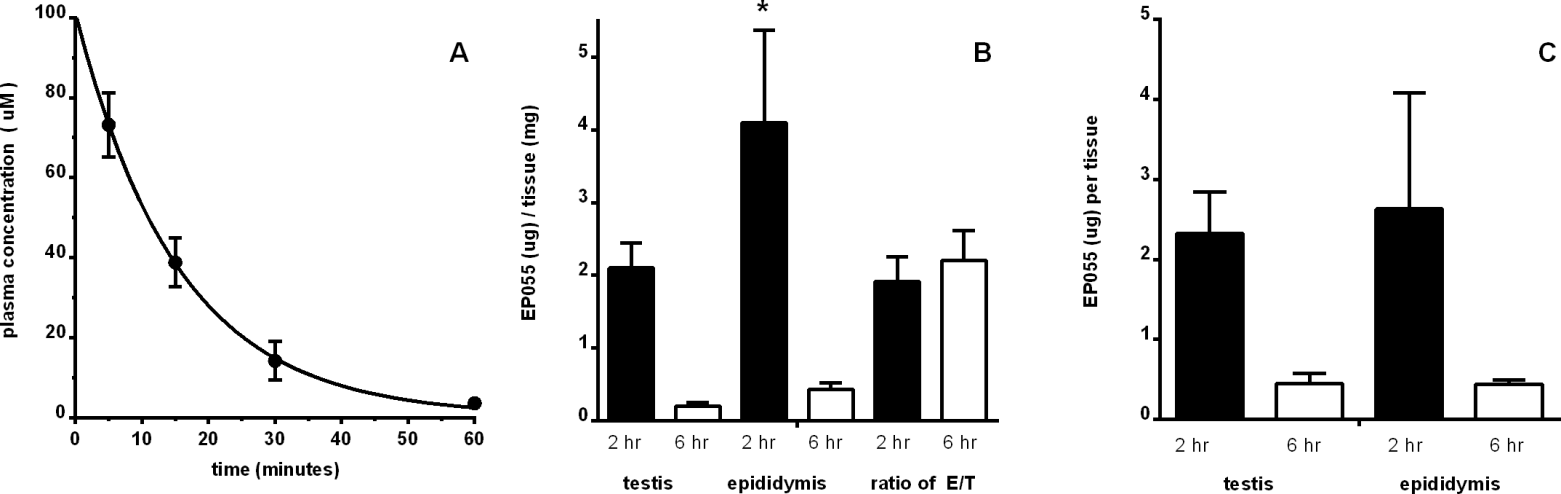
EP055 time course and tissue levels in *Macaca fascicularis* after a single i.v. injection. (A) Plasma half-life determination after infusion of a single dose (n = 3 males; 1mg/kg). t_1/2_ = 10.6 ± 2 minutes. (B) Testis tissue levels (n = 2 males per time point; 63.25 mg/kg) at 2 hr are statistically different from 2 hr epididymis levels (p < 0.05) by One-way ANOVA. The differences at 6 hr were not significant. There was no difference in the ratio of E/T at either time point. (C) Total μg of compound per tissue shows no significant difference between 2 hr levels and 6 hr levels (p < 0.05) by One-way ANOVA.

EP055 was tested in cynomolgus (*Macaca fascicularis)* males to determine its plasma half-life after intravenous (i.v.) infusion of a single dose and for binding to its target tissues. EPPIN, the target for EP055 binding, is an androgen dependent protein normally present only in the testis, epididymis and on spermatozoa [19]. Our initial study demonstrated a plasma half-life for EP055 of 10.6 minutes (Fig 2A). In a second study examination of macaque testis, epididymis, and plasma after i.v. infusion of a single dose of compound EP055 (63.25 mg/kg) demonstrated that EP055 was detected in testis and epididymis two hours and six hours post-infusion with significantly (p < 0.05) more EP055 (ug/mg of tissue) in the epididymis after 2 hours but not after 6 hours (Fig 2B). The ratio of EP055 present in the epididymis compared to the testis did not change significantly after 6 hours (Fig 2B). Because of a 1.78 weight differential between testis and epididymis (testis = 1.69 gm; epididymis = 0.95 gm) the total amounts of EP055 in each tissue were not significantly different at 2 and 6 hours (Fig 2C). To confirm the half-life, we calculated the initial plasma concentration and extrapolated the data to estimate that EP055 would have had a plasma half-life (t_1/2_) of 11.9 minutes in the target tissue experiment. This was consistent with our initial result (Fig 2A).

**Fig 3:**
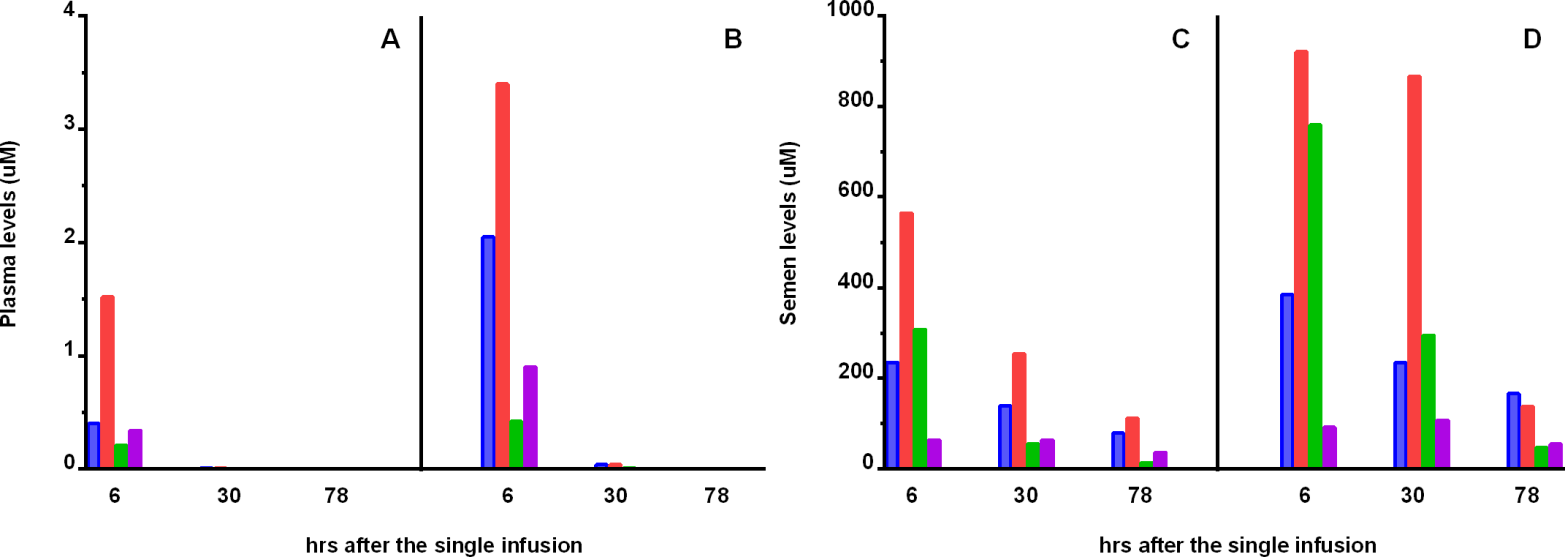
Levels of EP055 in individual *Macaca mulatta* males after a single i.v. dose. Each animal was given a low dose of EP055 (75–80 mg/kg). After sperm motility returned to normal, the same animal was given the high dose (125–130 mg/kg). Blue is male 28187, Orange is male 28043, Green is male 25854, Purple is male 28106. (A) Plasma and (C) Semen levels after the low dose of EP055. (B) Plasma and (D) Semen levels after the high dose of EP055.

As a result of these initial studies indicating the long-term retention of EP055 in the target tissues, we initiated a trial in rhesus (*Macaca mulatta)* males trained to provide semen samples without anesthesia to assess the availability of EP055 in semen and its effect on sperm motility. Four macaques were infused with a low dose (75–80 mg/kg) of EP055 followed by a recovery period of two weeks followed by a subsequent infusion of a high dose (125–130 mg/kg). EP055 was detected in plasma at 6–8 hours post-infusion and barely detectable at 28–30 hours post-infusion (Table 1; Fig 3A and 3B). In contrast, semen levels of EP055 were detectable up to 78 hours post-infusion (Table 1; Fig 3C and 3D).

**Table 1:** EP055 levels in rhesus macaque (Macaca mulatta) plasma and semen after infusion.

Infusion Level of EP055	Time after infusion	Plasma levels (uM)	Semen Levels (uM)	Ratio of S/P
Low dose	0	nd	nd	
(75–80 mg/kg)	6 hr	0.62 ± 0.3	# 293 ± 104	473
	30 hr	0.01 ± 0.0	128 ± 46	12800
	78 hr	nd	61 ± 22	
	14–18 days	nd	0.4 ± 0.3	
High Dose	0	nd	nd	
(125–130 mg/kg)	6–8 hr	0.97 ± 0.66	# 539 ± 186	556
	30 hr	0.02 ± 0.01	# 376 ± 168	18800
	78 hr	nd	101 ± 30	
	13–17 days	nd	0.9 ± 0.9	

EP055 levels shown are the mean ± SEM for 4 animals. (nd) is not detected.

# shows the significant increase in EP055 semen levels compared to untreated as in Fig 4A and 4B.

**Fig 4:**
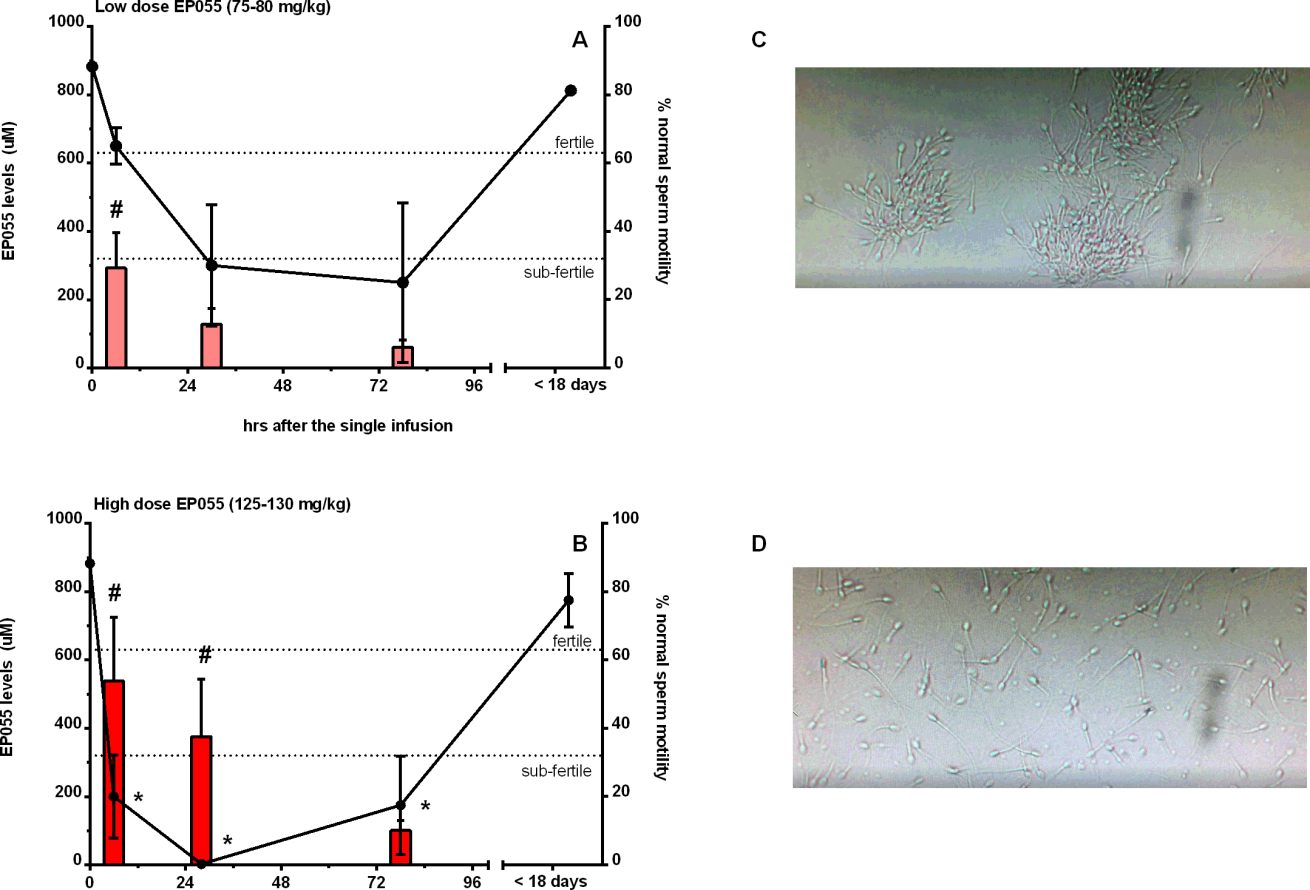
Effect of EP055 on rhesus macaque (*Macaca mulatta)* sperm parameters. Closed circles represent percent normal motility and bars represent semen levels of EP055 in A and B. (A) Correlation between EP055 semen levels and normal sperm motility after a single low dose (75–80 mg/kg) # show the significant increase in EP055 semen levels compared to untreated (Kruskal-Wallis analysis, p < 0.05). (B) Correlation between EP055 semen levels and normal sperm motility after a single high dose (125–130 mg/kg) # show the significant increase in EP055 semen levels and * show the significant decreases in motility by Kruskal-Wallis analysis (p< 0.05, compared to untreated). (C) Photograph of sperm from male 28043 at 28–30 hrs after the high dose showing clumping. See S1 Movie. (D) Photograph of sperm from male 28043 at high dose recovery (18 days post-infusion) showing normal spacing at the same sperm density. See S2 Movie. Results shown are for the mean ± SEM of n = 4 animals. Fertile and sub-fertile indices of motility are based on human clinical parameters [20].

Semen samples were collected approximately at 6, 30, and 78 hours post-infusion following the low and high dose treatments and weekly thereafter until complete recovery of normal motility (defined as >78%, F3+F4, see S1 Table). Samples of spermatozoa were examined to determine motility 30 minutes after semen collection to allow for clot liquefaction. As shown in Fig 4A, after low dose administration, normal sperm motility fell below 32% by 30 hours post-infusion, which is considered to be sub-fertile in humans [20]. Sperm motility in animal number 25854 was only slightly affected by the low dose treatment even though the levels of EP055 measured in the semen were not significantly different from the other three males (S2 Fig). However, sperm motility remained low in three out of four animals until at least 78 hours post-infusion; although not significantly different from untreated. Individual data for each animal are shown in S2 Fig. As shown in Fig 4B, after high dose administration, sperm motility fell significantly in all the animals to approximately 20% within 6 hours post-infusion; no normal motility was observed at 30 hours post-infusion. Recovery of sperm motility was obvious by 78 hours post-infusion, with significant full recovery in all animals by 18 days post-infusion. Sperm motility correlated with semen levels of EP055 following both the low (Fig 4A) and high (Fig 4B) dose infusion; with the exception of #25854 after a low dose infusion, (S2 Fig). Semen levels of EP055 increased significantly within 6 hours post-infusion relative to pre-treatment levels following both doses. In animals receiving the low dose, EP055 was still evident in semen at 30 and 78 hrs post-infusion, albeit at lower levels but not significantly different relative to 6 hours. Following high dose treatment, semen EP055 levels increased significantly relative to baseline and remained elevated between 6 and 30 hr. EP055 levels then declined, but were still evident in semen collected 78 hours post-infusion and barely detectable by 17 days (Table 1). The sperm motility data set for individual macaque males is given in S1 Table. Sperm motility post-infusion showed severe sperm clumping (Fig 4C) and many individual sperm with either no motility or with no progressive motility (Fig 4C). Normal motility was recovered after either low or high dose treatment and was indistinguishable from motility in control ejaculates (Fig 4D). Typical examples of the sperm motility after high dose treatment (Fig 4C) and the normal motility after recovery (Fig 4D) can be seen in the Supplemental videos. Monitoring of macaque blood and organ function during and after drug administration gave no indications of any side effects or health warning signs (S2 Table).

## Discussion

In searching for new non-hormonal male contraceptives, we assayed numerous small organic compounds that had multiple substitutions on a 1, 3, 5 triazine ring structure. One of these, EP055, which inhibited a-EAb from binding to EPPIN, inhibited human sperm motility, had a plasma half-life of 10–12 minutes, and was found to remain in the testis and epididymis for at least six hours (Fig 2). EP055 was subsequently tested in male macaques for its presence in semen and its effect on sperm motility in the ejaculates. As expected, the plasma levels of EP055 were barely detectable at 30 hours post-infusion (Table 1). However, our data demonstrate that infusion of EP055 resulted in the drug being retained in semen for up to 78 hours; effectively giving a potential contraceptive window of 24–48 hours following administration of the drug. The μM levels of EP055 measured in the semen (>300 μM; Table 1) at 6 and 30 hours after high-dose infusion far exceed the IC_50_ levels measured in human sperm CASA assays (199.5 μM). Human sperm CASA assays utilize spermatozoa from liquefied semen that have been separated from seminal plasma and washed through a gradient which changes the sperm surface environment as compared to epididymal sperm. The incubation time for human sperm in the CASA assay with a test compound is 1–2 hours. *In vivo* spermatozoa in the epididymis in the present macaque studies, which have never been exposed to seminal plasma, were exposed to EP055 for long periods of time that may have provided higher EP055 binding per spermatozoon. Our results indicate that higher affinity levels of drug binding to EPPIN in *in vitro* assays were not necessary for effective contraception.

Although low dose infusion of EP055 resulted in three out of four animals with marked loss of sperm motility, the lack of a motility response after 6 hours in animal number 25854 may indicate variations in drug metabolism or target availability. Most likely some level of target saturation on the sperm surface is necessary for the inhibition of progressive motility. High dose infusion of EP055 resulted in an inhibition of sperm motility in every animal, demonstrating a potentially strong contraceptive effect. Although three weeks was sufficient for the percent motility to return to normal, we did note after completion of the data analysis that more than 3 weeks was necessary for the velocity to recover completely.

All of the macaques remained healthy throughout the course of treatment and none of the parameters measured (S2 Table) showed clinically significant changes. However we did note that ALT (alanine aminotransferase) and glucose levels rose during treatment. Although the ALT values were within the normal range for rhesus macaques (18.9–94.2 U/l) the increase most likely indicates that the drug is processed in the liver or impacts liver function in some way. The glucose levels also increased, which often is the case during excitement, and under our experimental protocol, blood was drawn immediately after ejaculation and juice rewards. Excitement, as in the case of glucose, can affect both neutrophils and lymphocytes in what is called “physiologic neutrophilia” [21]. This is a shift in the cells found in the bloodstream versus cells in the tissues. Neutrophil numbers are within the reference range (2.4–9.6 x 10^3^/mm^3^), and slightly above the high end of the reference range after the high dose at 6 hrs; however it was not considered a clinical concern. Overall, EP055 had no long- term effects on any of the animals.

Men have only two practical choices for contraception; the condom which has a high typical use failure rate or vasectomy which is not readily reversible [1]. Our results demonstrate that the sperm surface protein EPPIN is a reasonable non-hormonal contraceptive target. Although EP055 has not yet been tested in a human contraceptive trial, our data indicate that it has a strong potential to be a male contraceptive that would provide a reversible, short-lived pharmacological alternative to condoms or vasectomy.

## Materials and methods

### Reagents

Chemicals were obtained from Thermo Fisher Scientific (Waltham, MA) unless noted. EP buffer: 21 mM HEPES, 94.6 mM NaCl, 4.5 mM KCl, 1.2 mM KH_2_PO_4_, 25 mM NaHCO_3_, 1.7 mM CaCl_2_, 1.2 mM MgSO_4_, 5.6 mM glucose, 22 mM sodium lactate, 0.7 mM sodium pyruvate. The final pH was adjusted to 7.30 just before use and supplemented with 0.4% BSA. Buffer was made fresh weekly, stored at 4°C and warmed before use. Phosphate buffered saline (PBS): 120 mM NaCl, 2.7 mM KCl, and 10 mM phosphate, pH = 7.6. Modified M16 was purchased from EMD Millipore (catalog number MR-016-D, Billerica, MA). EP012, EP054 and EP055 were synthesized by CiVentiChem (Research Triangle Park, NC). HEPES-buffered Tyrode's albumin lactate pyruvate (TALP-HEPES) medium containing 0.3% bovine serum albumin was made as described originally [22]. Concentrated stocks (100–600 mM) of EP012, EP054 and EP055 were made in DMSO and stored at -20°C between uses. Stocks were warmed to room temperature before use and made fresh every 4 weeks; freeze-thaw cycles were limited to 3–4. Aqueous dilutions of DMSO stocks were diluted into buffer within 2 hours of use. Pantoprazole sodium salt was provided by Toronto Research Chemicals (Toronto, Ontario, Canada). A working solution of pantoprazole sodium salt (3ng/mL in acetonitrile) was used as the internal standard spiking solution for LC-MS/MS analysis.

### AlphaScreen assay of a-EAb binding to EPPIN

The AlphaScreen® assay was carried as previously described [3]. Briefly, recombinant histidine-tagged recombinant EPPIN was pre-incubated with Ni-NTA chelate donor beads (catalog number AS101, Perkin Elmer, Waltham MA) and anti-Eppin 007 antibody [11,23] was pre-incubated with Protein A acceptor beads (catalog number 6760136, Perkin Elmer, Waltham MA) for 30 minutes. Equal volumes of donor and acceptor bead mixtures were pipetted into white opaque 384-well microplates (OptiPlate-384; PerkinElmer, Waltham, MA) in a final volume of 30 μL. Plates were covered with top seal and transferred to a Synergy 2 Multiplatform automated plate reader (Biotek, Winooski, VT). After shaking for 2 min, plates were read with an excitation 680/30 filter, an emission 570/100 filter and data acquired using Gen5 software (Biotek). Each set of samples was pipetted in 4 replicates. The final concentration of assay components was 58 nM EPPIN, 2 nM anti-Eppin 007 antibody, 5 μg/ml donor beads and 10 μg/ml acceptor beads in 0.1M Tris pH = 8, 0.1% bovine serum albumin and 0.01% Tween-20. Negative controls were performed under the same conditions in the absence of EPPIN or antibody and in the presence of beads only. A specific signal for each time point was calculated by subtracting the background signal (obtained in the absence of EPPIN) from its respective total signal. Anti-EPPIN 007 antibody binds to the SEMG1 binding site on the C-terminus of Eppin [11]. Non-specific interactions of compound with assay components were measured at the same time with the IgG detection kit (0.5 mM biotinylated rabbit IgG) to generate the bead interaction: 10 μg/ml donor and acceptor beads. (catalog number 6760617C, Perkin Elmer).

### Preparation of human spermatozoa and analysis of human sperm motility

Human semen samples were obtained from the Department of Obstetrics and Gynecology, University of North Carolina Memorial Hospital, Chapel Hill, NC. The Institutional Review Board determined that studies with these samples did not constitute human subjects research as defined under U.S. federal regulations [45 CFR 46.102 (d or f) and 21 CFR 56.102(c) (e) (l)] and the need for informed consent was waived. Semen samples were allowed to liquefy for 30 minutes and subjected to standard semen analysis to determine acceptability before freezing. All samples were stored in liquid nitrogen, and de-identified by the Department of Obstetrics and Gynecology, University of North Carolina Memorial Hospital, Chapel Hill, NC before further processing. A density gradient (Isolate catalog number 99264, Irving Scientific, Irving CA, or SpermCare catalog number 2221 and 2222, InvitroCare Inc., Frederick MD) was used to prepare spermatozoa for further analysis [18]. At least 3 independent experiments were done per assay, each using a single donor.

#### Computer assisted sperm analysis

Computer Assisted Sperm Analysis (CASA) was conducted as previously described [17]. Briefly sperm were washed after the density gradient with EP buffer-0.4% BSA and resuspended in modified M16 for compound treatments. Sperm were diluted to 5 x 10^6^/mL in 12x75 mm glass tubes with the appropriate treatment and incubated at 37°C in 5% CO_2_. For CASA analysis, sperm were loaded onto slides (CellVison 4 chamber (20 μM), catalog number CV-1020-4CV, Fertility Technology Resources, Murphy NC) and warmed to 37°C before analysis. Units for CASA parameters are as follows: VAP (average path velocity), VSL (straight line velocity), and VCL (curvilinear velocity) = μm/sec (microns/sec); ALH (lateral head amplitude) = μm; BCF (beat cross-frequency) = Hz. The relative mobility index (RMI) is a measure of the effect of compound on sperm and is calculated as the VSL x %normal motility and normalized to the appropriate DMSO control.

### Statistical analysis

*Sample size and power analysis was performed (*https://www.dssresearch.com/KnowledgeCenter/toolkitcalculators) *to determine the minimum number of males necessary to determine whether a given dose of the compound inhibits sperm motility relative to that in untreated males (baseline data)*. *For the primary outcomes listed above*, *a one-tailed test was used (either the compound will be inhibitory or have no effect) because it is unlikely that the compound will increase sperm motility*. *Based on prior experience with assessing sperm motility*, *the percentage of motile sperm varies among males*, *and can range from 0% to 95% depending upon the treatment*. *In normal males*, *motility ranges between 80–95%*. *Since the percentage motility post-treatment with this new compound is unknown*, *and since the motility can vary over time post-treatment*, *with a minimum of 4 males per group serving as their own controls for baseline sperm data*, *we have a power of 0*.*80 at α = 0*.*05 to detect a difference in percentage sperm motility and to derive statistically relevant data from the first study using this compound in nonhuman primate males*.

Graphing and statistical analysis was done using Prism version 6.07 Software (www.graphpad.com, La Jolla, CA). Results are expressed as the mean ± SEM. and (n) indicates the number of experiments. EC_50_ values were calculated from the best fit non-linear regression on transformed x = log(x) and normalized data. Half-life was calculated using the best-fit values for One-phase decay non-linear regression. One-way ANOVA was done with a Sidak post-test to correct for multiple comparisons (Fig 2). Non-parametric Kruskal-Wallis analysis was done with a Dunn post-test to correct for multiple comparisons (Fig 4). Statistical significance is indicated by * or # using p < 0.05.

### Ethical approval

The studies were conducted according to the National Institutes of Health Guide for the Care and Use of Laboratory Animals. All protocols were approved by ONPRC's Institutional Animal Care and Use Committee. The ONPRC protocol number is IP00000046 (1036: Contraceptive testing for semen availability in male monkeys).

### Animals, semen collection and semen analysis

The general care and housing of rhesus macaque monkeys (*Macaca mulatta*) was provided by the Division of Comparative Medicine, Oregon National Primate Research Center (ONPRC) as previously described [24,25]. Animals were pair-caged in a temperature-controlled (22°C) light-regulated (12L:12D) room and fed monkey chow twice a day, water *ad libitum* and fresh fruit or vegetables daily. Environmental enrichment in the form of toys, food puzzles, or other manipulanda were provided continuously by the Behavioral Science Unit at ONPRC, and changed weekly. All housing, feeding and environmental enrichment follow regulations required by the United States Department of Agriculture (USDA) Animal Welfare Act, the Office of Laboratory Animal Welfare, and outlined in the National Research Council, Institute for Laboratory Animal Resources, Guide for the Care and Use of Laboratory Animals.

The physical and behavioral condition of animals are monitored daily at ONPRC. Additional monitoring by Division of Comparative Medicine and/or clinical veterinary staff was provided immediately, at 1 hr, 2 hr and 6 hr post-administration of the contraceptive compound, and daily for up to 30 days after the final drug injection. Criteria used to assess animal health and welfare include: stool and urine quality and quantity, appetite and attitude (bright and alert, quiet, lethargic), pain (not eating, not moving, hunched over, lethargy/disinterest), vomiting, diarrhea, nausea, dehydration (unkempt coat, lack of urine production), and signs of wounds of injury. In addition, the ONPRC Standardized Pain Scoring system is used post-surgically to monitor an animal’s progress and improvement over time. Major criteria include animal appearance, posture, activity (including stool and urine production), vocalization and atypical behaviors.

Animals assigned for semen collection were evaluated on the basis of ease of restraint, number of attempts required to obtain a sample, and a qualitative judgment regarding the animal's tolerance of the procedure. If an animal proved uncooperative or a sample was not obtained after attempts on three separate occasions, it was rejected from the program. Remaining candidates were then accepted or rejected on the basis of a qualitative analysis of their sperm samples. Only monkeys with sperm numbers greater than 100 million per ejaculate and with more than 70% motile cells with normal morphology were acceptable.

Four, adult male rhesus macaques (*Macaca mulatta; mean age of 7*.*8 years of age*, *range* 7–10 years; mean body weight of 10.1 kg, range 8.1–13. 6 kg) successfully underwent pole and collar training and were acclimated for temporary chair restraint to provide semen samples via penile electroejaculation as per standard operating procedures in the Division of Comparative Medicine at ONPRC [25]. The pole and collar system provides a method of restraining awake monkeys for procedures that are safe for both animals and staff. Males were acclimated to wearing a titanium collar for one week, after which poles were attached to the collar to allow transfer of non-anesthetized animals to a nonhuman primate restraint chair for semen collection. Pole and collar training with subsequent training for chair restraint is done in stages using a positive reinforcement system that typically takes 6 weeks, but the training interval depends upon the individual animal. The duration of chair restraint at the times of semen collection was 30 minutes or less. Positive reinforcement in the form of fruit, vegetables, cereal or liquids was also administered during semen collections.

Semen was collected by direct penile stimulation with a P-T Electronics Model 303 PTE 110 Volt AC stimulator equipped with electrocardiogram pad electrodes (10–35 V, 20 ms duration, 18 pulses/sec) as previously described [25]. Semen was collected weekly for 3 weeks to establish baseline parameters for each male. Samples were inspected visually to determine sperm motility and suitability for the study. Sperm motility data from male 28106 presented in S1 Table is representative of pre-treatment samples. Immediately after collection, the seminal plug was separated from the liquefied fraction, placed into a cryovial, flash frozen in liquid nitrogen, and stored at -80C until evaluation for drug levels. Spermatozoa were prepared by centrifugation (7 min, 200 *g*) of the liquid portion of the ejaculate and resuspending the sperm pellet in TALP-HEPES medium containing 0.3% bovine serum albumin at 37°C. An aliquot was taken to determine concentration using a hemocytometer, percent motility and forward progression as previously described [25,26]. Baseline semen samples were collected from each male twice during the week prior to treatment. Semen was collected from each male 6–8 hr, 28–30 hr, and 78 hr post-infusion of low dose EP055 (75–80 mg/kg body weight) intravenously. After a one-week recovery period, a baseline semen sample was obtained, and the following week semen samples were collected as described above after an intravenous infusion of high dose EP055 (125–130 mg/kg).

Animals were returned to the ONPRC colony after the experiments were completed.

## Supporting information

S1 Fig Parent structure of the basic 1, 3, 5 triazine ring from which the lead compounds were derived. For EP012: R1 = CH_2_COOH; R2 = COOCH_3_; R3 = NHCOOCH_3_; R4 = OH. For EP054: R1 = CH_2_COOH; R2 = COOCH_3_; R3 = NHCOCH_3_; R4 = OCOCH_3_ For EP055: R1 = CH_2_COOH; R2 = COOCH_3_; R3 = NHCOCH_3_; R4 = OH.
(TIF)

S2 Fig Effect of EP055 on sperm parameters for individual *Macaca fascicularis* males. (A) #28187, blue, (B) #28043, orange, (C) #25854, green (D) #28106, purple. The top row of panels shows the correlation between EP055 semen levels and normal sperm motility after a single low dose (75–80 mg/kg). The middle row of panels shows the correlation between EP055 semen levels and normal sperm motility after a single high dose (125–130 mg/kg). The bottom row of panels shows the effect of a single high dose of EP055 on the curvilinear velocity (microns/sec) of spermatozoa in categories F1-F4, see S1 Table.
(TIF)

S1 Table Sperm motility data for individual macaque males treated with EP055. Spermatozoa were examined 30 minutes after collection. F4: frenzied movement, good progression, F3: rapid movement, good progression, F2: some movement, moderate forward progression, F1: some movement, absence of forward progression. F3+F4 are considered normal motility [24,25]. Normal samples (26028, 26848 and 24583) were from proven breeders used in the ONPRC in vitro fertilization core facility. Low dose is 75–80 mg/kg, High dose is 125–130 mg/kg).
(DOCX)

S2 Table Concentrations of blood constituents in male rhesus monkeys prior to and after infusion with low or high dose EP055. S2 Table lists salient values from serum biochemistry and hematological determinations in untreated males prior to, as well as 6–8 hr, 28–30 hr and 78 hr after infusion of low (75–80 mg/kg) or high (125–130 mg/kg) dose EP055. General indices of circulating glucose and ions, renal function (creatinine), liver function (e.g. total protein, alanine aminotransferase, g-glutamyl transferase), muscle function (e.g. aspartate aminotransferase) and blood cell constituents (e.g. erythrocytes) or any other parameters typically measured revealed no clinically significant difference between baseline levels and those obtained after either low or high dose EP055. Values represent the mean ±SEM for 4 animals in each group. Abbreviations: ALT alanine aminotransferase, AST aspartate aminotransferase, GGT g-glutamyl transferase, GLUC glucose, BUN blood urea nitrogen, CREAT creatine, MCV mean corpuscular volume, MHC mean corpuscular hemoglobin, MCHC Mean corpuscular hemoglobin concentration.
(DOCX)

S1 Movie Spermatozoa showing clumping behavior from animal 28043 at 28 hours after a high dose of EP055 (125–130 mg/kg). Also see S1 Table.
(MP4)

S2 Movie Spermatozoa showing normal swimming behavior from animal 28043 after recovery from a high dose of EP055 (125–130 mg/kg). Also see S1 Table.
(MP4)

S1 Additional Methods Video analysis for rhesus macaque curvilinear velocity, determination of EP012 half-life in rats, determination of EP054 and EP055 half-life in cynomolgus macaques, effect of EP055 on cynomolgus male reproductive system, standards and sample preparation for LC-MS/MS, I.V. infusion for rhesus macaque males and associated References. (DOCX)

S1 File NC3Rs ARRIVE guidelines checklist. (PDF)
