# Supplementary figures and images for: Inhibition of sperm motility in male macaques with EP055, a potential non-hormonal male contraceptive
Source: PLoS One. 2018 Apr 19;13(4):e0195953. doi: 10.1371/journal.pone.0195953 (PMC5908160; doi:10.1371/journal.pone.0195953)

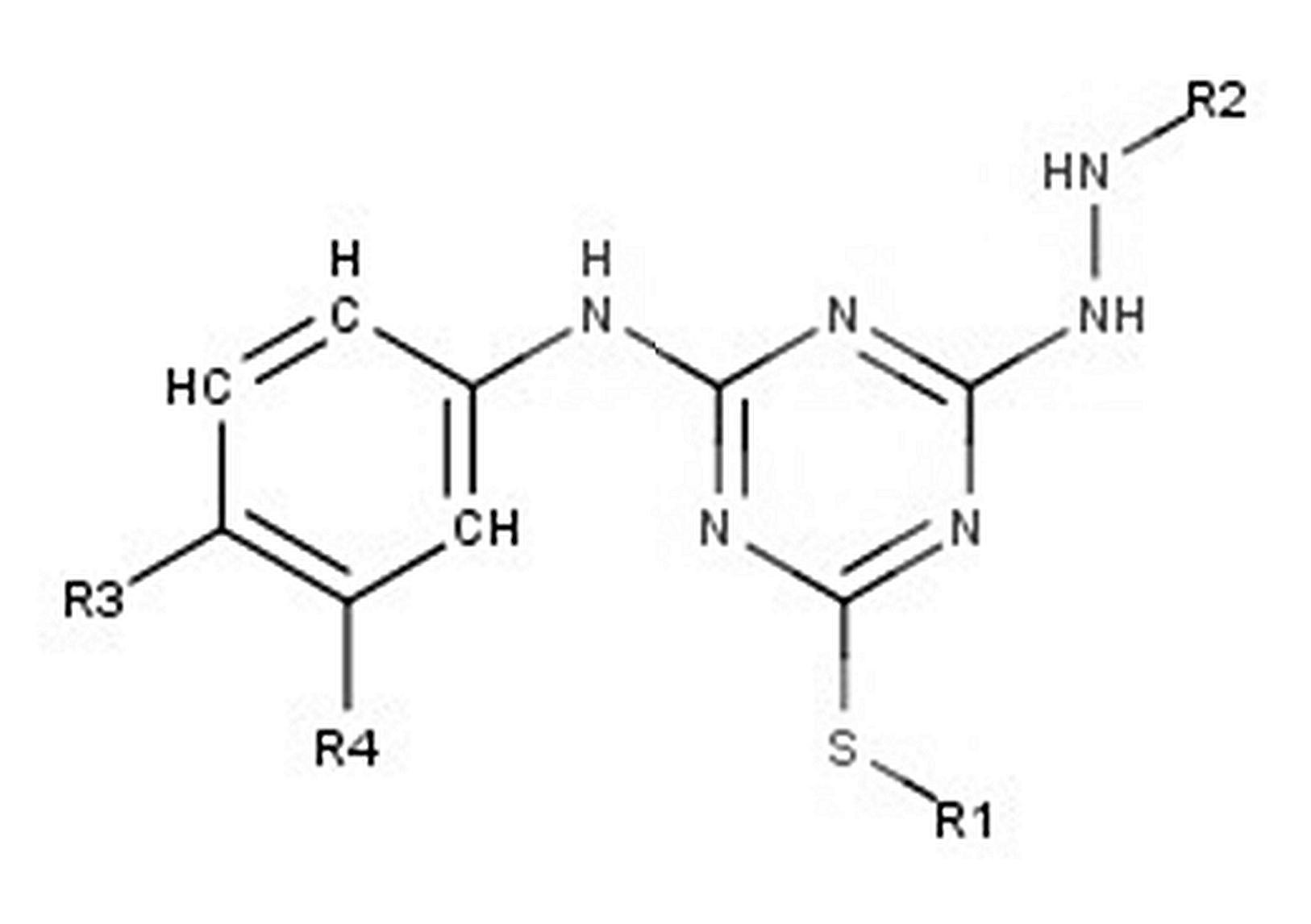

Supplement: S1 Fig — For EP012: R1 = CH2COOH; R2 = COOCH3; R3 = NHCOOCH3; R4 = OH. For EP054: R1 = CH2COOH; R2 = COOCH3; R3 = NHCOCH3; R4 = OCOCH3 For EP055: R1 = CH2COOH; R2 = COOCH3; R3 = NHCOCH3; R4 = OH. (TIF) [file pone.0195953.s001.tif]
