# Supplementary material for: Inhibition of sperm motility in male macaques with EP055, a potential non-hormonal male contraceptive
Source: PLoS One. 2018 Apr 19;13(4):e0195953. doi: 10.1371/journal.pone.0195953 (PMC5908160; doi:10.1371/journal.pone.0195953)
Supplement: S1 Table — Spermatozoa were examined 30 minutes after collection. F4: frenzied movement, good progression, F3: rapid movement, good progression, F2: some movement, moderate forward progression, F1: some movement, absence of forward progression. F3+F4 are considered normal motility [24,25]. Normal samples (26028, 26848 and 24583) were from proven breeders used in the ONPRC in vitro fertilization core facility. Low dose is 75–80 mg/kg, High dose is 125–130 mg/kg). (DOCX) [file pone.0195953.s003.docx]

| Animal |  | Date | Hrs post-iv | F3+F4 | F2 | F1 | Static | Clumps | VCL ± SEM (um/sec) |
| --- | --- | --- | --- | --- | --- | --- | --- | --- | --- |
| 26028 | Normal | 2/12/17 |  | 85% |  | 5% | 10% |  | 95.9 ± 4.5 |
| 26848 | Normal | 2/22/17 |  | 90% |  | 5% | 5% |  | 201.4 ± 7.1 |
| 24583 | Normal | 12/12/16 |  | 85% | 5% |  | 10% |  | 201.3 ± 7.0 |
| 28187 | Low dose | 10/24/16 | 6 | 65% |  | 35% |  | Few |  |
|  |  | 10/25/16 | 30 | 10% |  |  | 90% | Yes |  |
|  |  | 10/27/16 | 78 | 5% |  | 95% |  | Yes |  |
|  |  | 11/17/16 | 582 | 5% |  | 95% |  |  |  |
|  |  | 11/21/16 | 678 | 80% |  |  | 20% |  | 109.4 ± 13.8 |
|  | High dose | 11/29/16 | 8 | 30% | 50% | 10% | 10% | Yes | 52.9 ± 3.0 |
|  |  | 11/30/16 | 30 |  | 80% | 10% | 10% | Yes | 40.5 ± 2.7 |
|  |  | 12/2/16 | 78 |  |  | 95% | 5% | Fewer | 25.4 ± 2.6 |
|  |  | 12/16/16 | 414 | 85% |  | 10% | 5% | Few | 87.3 ± 6.2 |
| 28043 | Low dose | 11/8/16 | 6 | 75% |  |  | 25% | Few |  |
|  |  | 11/9/16 | 30 | 30% |  | 20% | 50% | Yes |  |
|  |  | 11/11/16 | 78 |  | 50% | 20% | 30% | Yes |  |
|  |  | 11/28/16 | 480 | 80% |  |  | 20% |  |  |
|  |  | 12/5/16 | 648 | 80% |  | 5% | 15% |  |  |
|  | High dose | 12/13/16 | 6 | 50% |  | 25% | 25% | Yes |  |
|  |  | 12/14/16 | 28 | <1% |  | 99% |  | Yes | 72.1 ± 12.4 |
|  |  | 12/16/16 | 78 | 60% |  | 10% | 30% | Few | 117.5 ± 16.3. |
|  |  | 12/28/16 | 360 | 65% |  | 10% | 25% |  |  |
|  |  | 12/30/16 | 408 | 70% |  | 5% | 25% | Yes |  |
|  |  | 1/4/17 | 528 | 80% | 5% |  | 15% |  | 135.0 ± 12.6 |
| 25854 | Low dose | 11/15/16 | 6 | 70% |  | 10% | 20% | Few |  |
|  |  | 11/16/16 | 30 | 80% |  | 5% | 15% | Fewer |  |
|  |  | 11/18/16 | 78 | 95% |  | <1% | 5% | Yes |  |
|  |  | 1/4/17 | 1206 | 83% |  | 2% | 15% |  |  |
|  |  | 1/6/17 | 1254 | 80% |  |  | 20% |  |  |
|  | High dose | 1/10/17 | 6 |  | 80% | 5% | 15% |  | 104.6 ± 6.9 |
|  |  | 1/11/17 | 30 |  | 60% | 10% | 30% | Yes | 120.9 ± 4.8 |
|  |  | 1/13/17 | 78 |  | 25% | 20% | 55% | Few | 94.2 ± 5.0 |
|  |  | 1/23/17 | 318 | 10% | 20% | 20% | 50% |  |  |
|  |  | 1/26/17 | 390 | 70% |  | 10% | 20% |  | 93.2 ± 5.0 |
|  |  | 2/2/17 | 558 | 90% |  | 1% | 9% |  |  |
| 28106 | Low dose | 2/3/17 | 0 | 90% | 3% |  | 7% |  | 127.8 ± 4.1 |
|  |  | 2/7/17 | 6 | 50% |  | 20% | 30% | Yes | 135.8 ± 6.8 |
|  |  | 2/8/17 | 30 | 5% | 45% | 25% | 25% | Yes | 105.1 ± 7.0 |
|  |  | 2/10/17 | 78 |  | 40% | 30% | 30% | Yes | 89.7 ± 6.3 |
|  |  | 2/21/17 | 342 | 80% |  | 5% | 15% |  | 105.0 ± 5.8 |
|  | High dose | 2/28/17 | 6 |  | 25% | 55% | 20% | Yes | 72.8 ± 4.8 |
|  |  | 3/1/17 | 30 |  | 5% | 20% | 75% | Yes | 67.5 ± 7.9 |
|  |  | 3/3/17 | 78 | 10% | 20% | 15% | 55% | Yes | 80.3 ± 6.9 |
|  |  | 3/13/17 | 318 | 25% | 20% | 30% | 25% | Yes |  |
|  |  | 3/16/17 | 390 | 50% | 10% | 15% | 25% | Yes |  |
|  |  | 3/23/17 | 558 | 15% | 40% | 15% | 30% | Yes |  |
|  |  | 4/6/17 | 894 | 78% |  | 2% | 20% |  | 101.7 ± 4.1 |
