# Supplementary material for: Inhibition of sperm motility in male macaques with EP055, a potential non-hormonal male contraceptive
Source: PLoS One. 2018 Apr 19;13(4):e0195953. doi: 10.1371/journal.pone.0195953 (PMC5908160; doi:10.1371/journal.pone.0195953)
Supplement: S2 Table — lists salient values from serum biochemistry and hematological determinations in untreated males prior to, as well as 6–8 hr, 28–30 hr and 78 hr after infusion of low (75–80 mg/kg) or high (125–130 mg/kg) dose EP055. General indices of circulating glucose and ions, renal function (creatinine), liver function (e.g. total protein, alanine aminotransferase, g-glutamyl transferase), muscle function (e.g. aspartate aminotransferase) and blood cell constituents (e.g. erythrocytes) or any other parameters typically measured revealed no clinically significant difference between baseline levels and those obtained after either low or high dose EP055. Values represent the mean ±SEM for 4 animals in each group. Abbreviations: ALT alanine aminotransferase, AST aspartate aminotransferase, GGT g-glutamyl transferase, GLUC glucose, BUN blood urea nitrogen, CREAT creatine, MCV mean corpuscular volume, MHC mean corpuscular hemoglobin, MCHC Mean corpuscular hemoglobin concentration. (DOCX) [file pone.0195953.s004.docx]

|  |  | Post Low Dose | | | | Post High Dose | | |
| --- | --- | --- | --- | --- | --- | --- | --- | --- |
|  | **Untreated** | **6-8 hrs** | **28-30 hrs** | **78 hrs** | **Recovery** | **6-8 hrs** | **28-30 hrs** | **78 hr** |
| **Blood constituents** | **Mean ± SEM** | **Mean ± SEM** | **Mean ± SEM** | **Mean ± SEM** | **Mean ± SEM** | **Mean ± SEM** | **Mean ± SEM** | **Mean ± SEM** |
| Total Protein (g/dl) | 6.7 ± 0.1 | 7.5 ± 0.0 | 7.7 ± 0.2 | 7.6 ± 0.1 | 7.1 ± 0.1 | 7.6 ± 0.2 | 7.8 ± 0.2 | 7.8 ± 0.1 |
| Albumin (g/dl) | 4.1 ± 0.0 | 4.7 ± 0.0 | 4.8 ± 0.1 | 4.7 ± 0.1 | 4.3 ± 0.1 | 4.7 ± 0.1 | 4.7 ± 0.1 | 4.8 ± 0.1 |
| Alkaline Phosphatase (U/l) | 106.0 ± 13.5 | 129.8 ± 19.2 | 128.5 ± 12.5 | 125.3 ± 16.3 | 79.0 ± 2.3 | 94.0 ± 4.8 | 95.7 ± 3.6 | 101.5 ± 8.7 |
| ALT (U/l) | 32.8 ± 1.3 | 44.8 ± 2.0- | 54.3 ± 3.8 | 60.3 ± 12.2 | 31.7 ± 2.3 | 49.7 ± 3.6 | 74.0 ± 10.8 | 53.0 ± 7.3 |
| AST (U/l) | 38.3 ± 2.7 | 49.8 ± 3.1 | 51.0 ± 6.6 | 73.8 ± 33.6 | 42.3 ± 2.6 | 68.3 ± 6.8 | 83.3 ± 14.3 | 30.0 ± 3.1 |
| GGT (U/l) | 63.0 ± 2.0 | 70.5 ± 2.1- | 72.5 ± 3.9 | 65.3 ± 2.7 | 62.3 ± 1.8 | 71.0 ± 1.0 | 71.3 ± 3.4 | 69.3 ± 3.8 |
| Total Bilirubin (mg/dl) | 0.1 ± 0.0 | 0.2 ± 0.1 | 0.2 ± 0.0 | 0.2 ± 0.0 | 0.2 ± 0.0 | 0.2 ± 0.1 | 0.2 ± 0.1 | 0.1 ± 0.0 |
| GLUC (mg/dl) | 46.8 ± 4.5 | 89.0 ± 6.7 | 90.0 ± 10.3 | 91.5 ± 7.4 | 58.3 ± 4.5 | 101.3 ± 10.2 | 92.0 ± 9.7 | 85.0 ± 8.7 |
| BUN (mg/dl) | 16.5 ± 1.8 | 17.2 ± 1.9 | 15.0 ± 1.5 | 18.8 ± 3.2 | 16.5 ± 1.9 | 18.8 ± 2.3 | 15.3 ± 2.4 | 18.3 ± 2.2 |
| CREAT mg/dl) | 0.8 ± 0.0 | 1.0 ± 0.0 | 1.0 ± 0.0 | 1.0 ± 0.0 | 0.8 ± 0.0 | 1.0 ± 0.1 | 1.0 ± 0.0 | 1.0 ± 0.0 |
| Potassium (mEq/l) | 3.6 ± 0.1 | 3.9 ± 0.2 | 4.2 ± 0.2 | 4.0 ± 0.1 | 4.0 ± 0.1 | 4.0 ± 0.1 | 4.0 ± 0.1 | 4.4 ± 0.2 |
| Sodium (mEq/l) | 146.3 ± 0.8 | 149.0 ± 0.9 | 149.0 ± 1.1 | 147.0 ± 1.2 | 146.7 ± 0.3 | 147.7 ± 0.6 | 149.0 ± 0.0 | 149.5 ± 1.2 |
| Chloride (mEq/l) | 108.3 ± 1.0 | 109.3 ± 1.5 | 110.3 ± 0.9 | 108.0 ± 0.8 | 107.0 ± 0.0 | 108.7 ± 0.3 | 108.7 ± 0.6 | 108.5 ± 0.9 |
| Magnesium (mg/dl) | 1.8 ± 0.0 | 1.8 ± 0.0 | 1.7 ± 0.1 | 1.8 ± 0.0 | 1.9 ± 0.1 | 2.2 ± 0.1 | 1.9 ± 0.0 | 1.9 ± 0.1 |
| Phosphorus (mg/dl) | 4.8 ± 0.4 | 5.3 ± 0.3 | 4.0 ± 0.5 | 4.1 ± 0.6 | 5.1 ± 0.2 | 5.2 ± 0.4 | 3.2 ± 0.6 | 4.2 ± 0.8 |
| Cholesterol (mg/dl) | 140.0 ± 16.5 | 154.5 ± 19.0 | 149.5 ± 19.5 | 144.0 ± 17.0 | 138.7 ± 19.3 | 146.3 ± 18.1 | 147.3 ± 23.4 | 152.0 ± 19.4 |
| Triglycerides (mg/dl) | 44.0 ± 5.9 | 63.8 ± 5.7 | 75.3 ± 28.9 | 57.3 ± 14.1 | 38.0 ± 6.9 | 84.3 ± 19.8 | 59.3 ± 18.5 | 68.0 ± 10.6 |
| Leukocytes (x 10^3^/mm^3^) | 7.0 ± 1.7 | 12.8 ± 2.7 | 11.8 ± 3.1 | 11.2 ± 3.3 | 5.9 ± 1.0 | 12.3 ± 1.8 | 7.7 ± 1.4 | 9.7 ± 2.3 |
| Neutrophils (x 10^3^/mm^3^) | 3.4 ± 1.2 | 9.5 ± 2.0 | 7.2 ± 2.0 | 6.6 ± 2.2 | 4.5 ± 1.7 | 10.3 ± 1.4 | 5.1 ±0.8 | 5.4 ± 1.1 |
| Lymphocytes (%) | 46.1 ± 5.1 | 18.8 ± 1.9 | 31.1 ± 4.8 | 33.7 ± 5.0 | 44.8 ± 5.4 | 21.6 ± 9.9 | 32.4 ± 4.3 | 34.6 ± 4.5 |
| Monocytes (%) | 6.1 ± 0.9 | 4.9 ± 0.1 | 5.7 ± 0.3 | 5.5 ± 0.5 | 6.4 ± 0.2 | 5.2 ± 0.4 | 5.5 ± 0.6 | 5.6 ± 2.3 |
| Eosinophils (%) | 1.7 ± 0.3 | 1.2 ± 0.3 | 1.4 ± 0.1 | 2.0 ± 0.5 | 1.4 ± 0.3 | 0.9 ± 0.1 | 1.4 ± 0.1 | 1.4 ± 4.5 |
| Basophils (%) | 0.5 ± 0.0 | 0.6 ± 0.1 | 0.6 ± 0.1 | 0.6 ± 0.1 | 0.5 ± 0.1 | 0.5 ± 0.1 | 0.5 ± 0.1 | 0.5 ± 2.3 |
| Hematocrit | 41.7 ± 1.1 | 44.3 ± 1.0 | 43.9 ± 1.9 | 40.6 ± 0.8 | 42.4 ± 1.1 | 45.2 ± 1.0 | 45.9 ± 0.9 | 43.3 ± 4.5 |
| Hemoglobin (g/dl) | 13.6 ± 0.3 | 14.4 ± 0.3 | 14.4 ± 0.7 | 13.3 ± 0.3 | 13.8 ± 0.4 | 14.7 ± 0.4 | 15.0 ± 0.4 | 14.1 ± 2.3 |
| Erythrocytes (x 10^6^/mm^3^) | 5.6 ± 0.2 | 5.9 ± 0.2 | 5.9 ± 0.3 | 5.5 ± 0.2 | 5.7 ± 0.2- | 6.1 ± 0.2 | 6.1 ± 0.2 | 5.8 ± 4.5 |
| MCV (fl/cell) | 74.6 ± 1.2 | 75.1 ± 1.2 | 74.7 ± 1.0 | 74.6 ± 1.2 | 74.1 ± 0.9 | 74.5 ± 1.1 | 74.8 ± 1.0 | 74.9 ± 2.3 |
| MCH (pg/cell) | 24.3 ± 0.3 | 24.5 ± 0.3 | 24.4 ± 0.2 | 24.5 ± 0.3 | 24.1 ± 0.4 | 24.2 ± 0.4 | 24.4 ± 0.3 | 24.3 ± 4.5 |
| MCHC  (g/dl) | 32.6 ± 0.1- | 32.6 ± 0.1 | 32.7 ± 0.3 | 32.8 ± 0.1 | 32.5 ± 0.2 | 32.5 ± 0.1 | 32.7 ± 0.2 | 25.0 ± 7.5 |
| Platelets  (x 10^6^/mm^3^) | 230.3 ± 31.9 | 268.5 ± 38.7 | 185.8 ± 35.8 | 270.8 ± 25.8 | 295.7 ± 11.8 | 298.3 ± 39.0 | 301.7 ± 36.5 | 292.8 ± 48.1 |
