# Supplementary material for: Inhibition of sperm motility in male macaques with EP055, a potential non-hormonal male contraceptive
Source: PLoS One. 2018 Apr 19;13(4):e0195953. doi: 10.1371/journal.pone.0195953 (PMC5908160; doi:10.1371/journal.pone.0195953)
Supplement: S1 Additional Methods — (DOCX) [file pone.0195953.s007.docx]

# Supplementary Material & Methods

## Video analysis for rhesus macaque sperm curvilinear velocity

Sperm were diluted in TALP-HEPES medium containing 0.3% bovine serum albumin to achieve 50-100 sperm per field. Videos were taken using an iPhone 6s Plus positioned 12 inches from a Dell model E170Sc 17-inch LCD monitor (1280 x 1024 pixels at 60 Hz, 0.264 mm) attached to an Olympus CK40 inverted microscope using 20x magnification (10x objective). The video camera collected images at a resolution of 1080 x 1920 pixels at 30 frames per second and 401 ppi with a typical contrast ratio of 1300:1 for at least 8 seconds. The pixel size was 1.2 microns and the videos in .mp4 or .mov format. Videos were converted to .avi format using Any Video Converter (version 6.06, http://www.anvsoft.com/any-video-converter-free, Ultimate Media Solutions, AnvSoft Inc). These files were uncompressed using VirtualDub (version 1.10.2, <http://www.virtualdub.org/>).

Images were analyzed using the MTrackJ plug in (version 1.5.1, <https://imagescience.org/meijering/software/mtrackj/>) for ImageJ (1.51K, <https://fiji.sc/>) [1,2]. Briefly, files were imported as uncompressed .avi files, converted to greyscale and saved as .TIFF files. Each image was calibrated by measuring a representative sperm head (acrosomal apex to midpiece) using the Straight Edge Tool, using 5.18 microns as the standard length of a *Macaca mulatta* sperm head [3]. Approximately 50 sperm per video were manually tracked for the entire length of the track and the mean curvilinear velocity of each track calculated using the MEASURE function. Tracks used in the final analysis were a minimum of 0.4 seconds.

## Determination of EP012 half-life in rats

Preliminary half-life measurements for EP012 were carried out by Calvert Laboratories (Scott

Township, PA; Study No:0835RE37.001). Treatment of animals was in accordance with Calvert Standard Operating Procedures which adhere to the regulations outlined in the USDA Animal Welfare Act (9 CFR Parts 1, 2 and 3) and the conditions specified in the Guide for the Care and Use of Laboratory Animals ([4]). The Calvert Institutional Animal Care and Use Committee (IACUC) approved the study protocol prior to finalization to insure compliance with acceptable standard animal welfare and humane care.

The intravenous formulation was prepared by mixing an appropriate amount of EP012 (1 mg/kg) with an appropriate volume of intravenous vehicle (DMSO). The pH of the intravenous formulations was confirmed to be 7.4-7.6 and instilled through a syringe filter for sterilization prior to dosing. Three Sprague Dawley male rats (8-14 weeks; 300 to 325 g) were surgically implanted with a catheter in a jugular vein using polyurethane tubing and glycerol or dextrose solution as the lumen lock solution. The intravenous formulation was administered as a bolus injection into a lateral tail vein.

Serial blood samples (~0.30 mL each), were obtained from each animal via the jugular vein catheter at the following timepoints post-dose: 0.083, 0.167, 0.333, 0.5, 1, 2, 4, 8, 12, and 24 hours. Plasma (~125 µL per sample) was harvested following centrifugation of the chilled blood samples at 3000 RPM for 10 minutes at +4 °C. Derived plasma was transferred into prelabeled plastic vials and stored frozen at approximately -70 °C or lower until processing.

## Determination of EP054 and EP055 half-life in cynomolgus male macaques

Half-life measurements for EP054/EP055 were done by Sinclair Research Center, LLC (SRC) (Study No. S14109, Auxvasse, MO). This study was reviewed and approved by the Sinclair IUCAC committee and complied with all applicable sections of USDA Policy 12 and the Final Rules of the Animal Welfare Act regulation (9 CFR). This study used 3 naïve cynomolgus (*Macaca fascicularis*) males (3-4 yrs old, 4 kg or less). Doses of EP054 in 3% DMSO-PBS, pH 7.6 were infused by i.v. at 1.0 mg/kg. The infusion rate was 4 ml/min. Plasma samples were collected at 0, 5, 15, 30 min and 1, 4, 8 and 24 hours, and immediately frozen on dry ice. Samples were stored at -80 °C until processing.

## Effect of EP055 on the cynomolgus male macaque reproductive system

Evaluation of the effects of an i.v. infusion of EP055 on male cynomolgus macaque reproductive system was done by Sinclair Research Center, LLC. (Study No: S14574). This study was reviewed and approved by the Sinclair IUCAC committee and complied with all applicable sections of USDA Policy 12 and the Final Rules of the Animal Welfare Act regulation (9 CFR). This study used 6 naïve cynomolgus males (3-4 yrs old, 4 kg or less). Doses of EP055 in 5% DMSO-PBS, pH 7.6 or vehicle control were infused by i.v. at 63.25 mg/kg. The infusion rate was 4 ml/min. Plasma and tissues (epididymis and testis) were collected at 2 hr or 6 hr and immediately frozen on dry ice. Samples were stored at -80 °C until processing. Half-life was approximated using the 2 hr and 6 hr points and an estimation of the initial plasma level using the initial dose of 63.25 mg/mg, the circulating blood volume of 65 ml/kg [5], average hematocrit of 41.7 (S2 Table) and average animal size (3.72 kg for n=4).

## Standards and Sample preparation for LC-MS/MS

Sample preparation and LC-MS/MS analysis were done by OpAns LLC (Durham, NC). Plasma samples (both rat and macaque) were thawed at room temperature and processed by adding 160 µL of methanol (including 10 µL internal standard) to 25 µL of the sample. Samples were vortexed for 5 minutes, centrifuged at 2684 x g for 5 minutes. 50 µL of supernatant was removed for analysis by HPLC-MS/MS.

Semen or tissue samples were thawed at room temperature. PBS at pH 7.6 was added to each sample at a ratio of 2:1 and sonicated in an ice chilled bath for 5 minutes before homogenization using a Polytron unit (Kinematica AG, Lucerne, Switzerland). An aliquot (25 uL) of each sample homogenate was processed as described for plasma samples.

Calibration standards (Pantoprazole sodium salt), and EP012, EP054 or EP055 for the standard curve at five concentrations ranging from 1-1000 ng/ml were freshly prepared for each run. Calibrants and quality control samples were run before and after the corresponding test samples. All chromatograms from the analytical run were reviewed to verify that the appropriate peaks had been identified and correctly integrated. The concentrations of test compound were determined by using the weighted (1/x2) power regression analysis of the calibration standards using MassHunter Quantitative Analysis for QQQ. (Model 6460 Triple quadrupole mass spectrometer, Agilent Technologies Inc, Santa Clara CA)

HLPC was done using an Agilent 1200 series unit fitted with an ACE Excel 2 C18-AR, 3 x 50 mm column (Agilent Technologies Inc). The mobile phases were as follows: A) water with 0.1% ammonium fluoride, B) methanol with 0.1% ammonium fluoride. The samples were loaded in 85% A/15% B, and then washed with 5% A/95% B. The samples were eluted in 100% B followed by a wash in 85% A/15% B. The flow rate was 0.6 mL/min and the run-time was 3.8 minutes. HPLC-MS/MS data were acquired using the proprietary software application MassHunter Workstation Acquisition using an Agilent 6460 mass spectrophotometer. Data were integrated using the software application and calibration plots of area ratios versus each test article concentrations were constructed, and a 1/x2 weighted power regression was applied to the data using MassHunter Quantitative Analysis for QQQ. Descriptive statistics were performed using Excel 2013.

Initial analysis of EP054 in plasma revealed no compound present, even at the earliest timepoints because of complete hydrolysis to EP055. Therefore, samples were analyzed for the presence of EP055. These results are presented in Fig 2A.

## I.V. infusion of macaque males

Prior to infusion of EP055, each male was sedated with ketamine by the surgical staff at ONPRC and transported to the operating room. After positioning in dorsal recumbency, the male was intubated and placed under inhalant (isoflurane) anesthesia. Bilateral intravenous catheters were placed in cephalic veins, one for infusion and one for back-up. A percutaneous arterial line was placed in the right femoral artery for direct blood pressure measurement. Invasive blood pressure, heart rate, SpO_2_, and respirations were monitored by the surgical staff throughout the procedure. Initially, the low doses of EP055 in 5% DMSO-PBS 7.6 was infused by i.v. at 75-80 mg/kg, using a Medfusion 3500 pump (Smiths Medical, St. Paul, MN), and was started at a low flow rate (0.6mg/kg/min). In 3 of 4 males, a 5-10% increase in blood pressure and heart rate were noted within the first 2-3 minutes of infusion of EP055. In these cases, the infusion was temporarily suspended until these parameters subsequently declined to normal. The infusion rate was then doubled to 1.2 ml/kg/min after 5-10 minutes and the final volume of EP055 infusate was administered. Blood pressure and heart rate remained normal throughout the rest of the infusion interval. The total volume of EP055 infusate ranged from 72 ml – 136 ml, depending upon the weight of the animal, and the duration of infusion was 10-13 minutes. Following completion of the infusion, all animals recovered normally. Animals were monitored by clinical veterinary staff immediately, at 1 hr, 2 hr and 6-8 hr post-infusion, and daily for up to 30 days after the final infusion of EP055. No adverse effects were observed. Two weeks after the infusion of the low dose and collection of the next set of baseline semen samples, males were infused with the high dose of EP055 (125-130 mg/kg) as described above, and monitored similarly. Likewise, no adverse effects were observed after the high dose infusion.

Blood was collected from the brachial vein at the time of each semen collection, and used to determine complete blood counts and serum biochemistry tests (including electrolytes, glucose, lipids, proteins, enzymes and metabolic by-products) as performed in the Clinical Pathology Laboratory in the Division of Comparative Medicine at ONPRC.

# References

1. Schindelin J, Arganda-Carreras I, Frise E, Kaynig V, Longair M, Pietzsch T, et al. Fiji: an open-source platform for biological-image analysis. Nat Methods. 2012;9: 676–682. doi:10.1038/nmeth.2019

2. Schneider CA, Rasband WS, Eliceiri KW. NIH Image to ImageJ: 25 years of image analysis. Nat Methods. 2012;9: 671–675.

3. Tollner TL, Dong Q, VandeVoort CA. Frozen-thawed rhesus sperm retain normal morphology and highly progressive motility but exhibit sharply reduced efficiency in penetrating cervical mucus and hyualuronic acid gel. Cryobiology. 2011;62: 15–21. doi:10.1016/j.cryobiol.2010.11.004

4. National Research Council (US) Committee for the Update of the Guide for the Care and Use of Laboratory Animals. Guide for the Care and Use of Laboratory Animals [Internet]. 8th ed. Washington (DC): National Academies Press (US); 2011. Available: http://www.ncbi.nlm.nih.gov/books/NBK54050/

5. Wolfe-Coote S. The laboratory primate. Amsterdam ; Boston: Elsevier/Academic Press; 2005.
